# Photophysical Properties and Metal Ion Sensing of a Pyrene-Based Liquid Crystalline Dimer

**DOI:** 10.3390/ijms26062566

**Published:** 2025-03-13

**Authors:** Mihaela Homocianu, Elena Perju

**Affiliations:** “Petru Poni” Institute of Macromolecular Chemistry, 41A, Grigore Ghica Voda Alley, 700487 Iasi, Romania; elena.perju@icmpp.ro

**Keywords:** pyrene dimer, liquid crystals, solvatochromism, acidochromism, metal ion sensing, fluorescence spectroscopy

## Abstract

This study investigates the liquid crystalline behavior, photophysical properties, and metal ion sensing capabilities of a pyrene-based imine dimer (DPyH9). The compound exhibits monotropic nematic mesophase behavior, with a glass transition at 43 °C, as confirmed by polarized light microscopy (PLM) and differential scanning calorimetry (DSC). Its photophysical properties, including UV-vis absorption, solvatochromic fluorescence, and acidochromism, observed through spectral shifts upon HCl addition, were systematically analyzed. Notably, DPyH9 displayed selective metal ion sensing capabilities towards Sn^2+^ and Cu^2+^ with binding constants of 4.51 × 10^6^ M^−1^ and 4.03 × 10^7^ M^−1^ and detection limits of 1.61 × 10^−5^ M (Sn^2+^) and 4.73 × 10^−5^ M (Cu^2+^). Fluorescence titrations revealed distinct responses: Sn^2+^ induced an initial quenching and an enhancement at higher concentrations, while Cu^2+^ caused significant fluorescence quenching. These results therefore highlight DPyH9 as a potential candidate for sensing applications and optoelectronic devices.

## 1. Introduction

Luminescent materials exposed to external stimuli such as mechanical force, vapor, light, and/or temperature present modifications in their emission responses that are suitable for applications in chemosensors, photoelectric devices, and related fields [1,2,3]. The external stimuli could induce molecular stacking changes, leading to phenomena like aggregation-induced emission (AIE) or quenching, which are critical for optimizing fluorescence efficiency in both solution and aggregated states. Consequently, fluorescence efficiency in both solution and aggregation states represents an essential factor in designing luminescent materials [4,5]. Recent advances in pyrene-based fluorophores, for instance, have demonstrated their utility in detecting heavy metal ions (e.g., Ag⁺, Pb^2^⁺) through mechanisms like excimer–monomer switching and chelation-enhanced quenching [6]. Also, Liang et al. [7] developed a pyrene-based fluorescent sensor (PP) that initially exhibits weak fluorescence but enhances in the presence of Cu^2+^, enabling sensitive detection. It demonstrates high selectivity for Cu^2+^, with a detection limit of 0.036 μM, and effective senses for 3-nitropropionic acid under acidic conditions.

In 2022, Yeldir et al. [8] synthesized a monomer, 2-methoxy-6-((pyren-1-yl-imino)methyl)phenol (VP), which exhibited high sensitivity and selectivity as a fluorescence sensor for Sn^2^⁺ ions, with a low detection limit of 4.24 nM. Upon excitation at 320 nm, VP emitted a bright blue fluorescence at 445 nm in the presence of Sn^2^⁺, demonstrating its potential for metal ion detection.

Moreover, Rana et al. [9] prepared a novel pyrene-based Schiff base fluorophore, PY-SB, which exhibits significant potential as a selective “turn-on” sensor for Sn^2^⁺ ions, demonstrating remarkable aggregation-induced enhanced emission (AIEE) properties. This compound not only shows a 24-fold increase in fluorescence intensity in mixed solvents but also effectively detects Sn^2^⁺ ions with a detection limit of 5.4 µM and a binding constant of 2 × 10⁴ M⁻^1^. The sensing mechanism involves a photoinduced electron transfer (PET) process that is inhibited upon complexation with Sn^2^⁺, leading to enhanced fluorescence.

Among these materials, liquid crystals (LCs) have gained significant attention due to their unique ability to exist in both liquid and ordered states, making them suitable for applications such as use in advanced display technologies, nonlinear optical materials, and sensing applications [10,11]. Recent studies on pyrene-based LC dimers highlight their ability to form mesophases, including twist-bend nematic phases, driven by intermolecular π–π* interactions and molecular curvature. For example, nonsymmetric pyrene dimers with methylene-ether spacers exhibit glass-forming behavior and stable helical field lengths in the nanoscale range, enabling their use in stimuli-responsive devices [12]. An easy method to design functional LC materials is to use moieties and/or functional groups that can self-assemble by intra-/intermolecular interactions into various mesophases. Conjugated organic materials play an important role as active layers due to the delocalized electronic structure formed by bonding electron-donating and withdrawing groups from a π-electronic bridge with a large conjugated system. Among the π-conjugated planar aromatic units, pyrene derivatives have been widely investigated due to their excellent photoelectric properties, such as strong emission, efficient excimer formation, suitable intermolecular stacking, and self-assembly into lamellar and columnar mesophases [13]. Although the pyrene unit typically emits strongly in dilute solutions, it tends to form dimers (or excimers) through π–π* interactions in the aggregated state, resulting in spectral shifts and fluorescence quenching [14,15]. Ultrafast dynamics studies on pyrene dimers reveal two distinct excimer formation pathways mediated by intermolecular conical intersections, offering mechanistic insights into controlling emission behavior in aggregated systems [16]. Despite these advances, the integration into LC dimers for sensing applications remains underexplored, particularly in balancing mesomorphic stability with stimuli-responsive luminescence.

In addition to pyrene derivatives, azomethines have been widely studied because they are easy to synthesize and purify, exhibit liquid crystalline behavior, and possess favorable optoelectronic properties [15,17,18]. Additionally, the nitrogen heteroatom of the azomethine group can act as an acceptor unit for protons, leading to different molecular stacking, charge distribution, and luminescence intensity shifts.

The vitrification phenomenon leads to LC polymers; however, due to their high melt viscosity, it is difficult to obtain a fast and uniform response of the mesomorphic arrangement to external stimuli [19]. Instead, LC dimers with medium weights and/or multiple mesomorphic moieties often form a glassy liquid crystalline phase to suppress the crystallization [20]. Prior studies on pyrene-based metal–organic frameworks (MOFs) highlight how structural modifications can enhance sensing performance, suggesting similar potential for LC dimers [21]. Due to the temperature-dependent optoelectronic properties, the mesomorphic glass-forming compounds can be applied as nonlinear-optical materials [22].

In this study, we focus on a pyrene-based imine dimer (DPyH9) that combines liquid crystallinity with photophysical properties for potential sensing applications. Herein, we investigate the solvatochromic, acidochromic, and metal ion sensing properties of this compound. The liquid crystalline behavior of DPyH9 was characterized using PLM and DSC. The combination of liquid crystallinity with the photophysical properties within a single molecular entity offers a unique platform for the development of multifunctional materials with potential applications in sensing and other areas.

## 2. Results and Discussion

The pyrene-based imine dimer was prepared via a condensation reaction of 1,9-bis(4-formylphenoxy)-nonane with an excess of 1-aminopyrene in the presence of acetic acid as a catalyst to avoid the monosubstituted compound formation [15]. The phase transition behavior of the DPyH9 was thoroughly examined by PLM and DSC analyses under two cycle scans with similar heating–cooling rates. The transition temperatures recorded by both methods are presented in Table 1.

**Figure 1:**
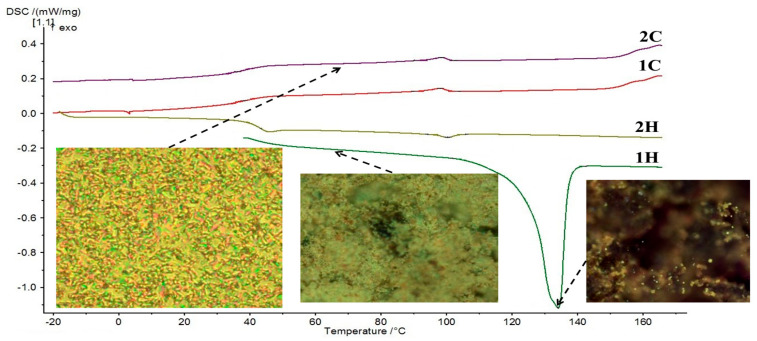
DSC traces and PLM photographs of DPyH9 using cross polarizers.

The DPyH9 presents a monotropic liquid crystalline behavior, with a fine granular nematic texture [23] that freezes as a mesomorphic glass [24] (photographs inserted in Figure 1) observed by PLM. This phase behavior was confirmed by DSC investigation, which showed in the first heating scan an endothermic peak at 134 °C corresponding to a crystalline–isotropic transition, and upon the second heating, a glass transition was observed at 43 °C, followed by an endothermic peak at 100 °C corresponding to a nematic–isotropic transition. In both cooling scans, one exothermic peak corresponding to an isotropic–nematic transition and a glass transition due to the frozen mesophase appeared in the same temperature range, highlighting the thermotropic behavior reproducibility of the dimer. As expected, the enthalpy of the nematic–isotropic and isotropic–nematic transitions compared to the crystalline–isotropic transition has lower values, which is in line with the lower energy requirements for the transition between two states with closer ordering degrees [25]. A series of symmetric pyrene-based liquid crystal dimers, which tend to be monotropic and form stable glass phases, have also been reported [26]. Furthermore, incorporating a pyrene unit into the molecular structure may not only hinder crystallization but also introduce new functionalities to the material, such as fluorescent properties [13]. Therefore, low-molar-mass liquid crystal glasses have been attracting considerable attention in the field of materials science due to both their fundamental interest for detailed studies of monotropic phases and their potential applications in electrooptic devices.

### 2.1. Photophysical Properties of the DPyH9

**Figure 2:**
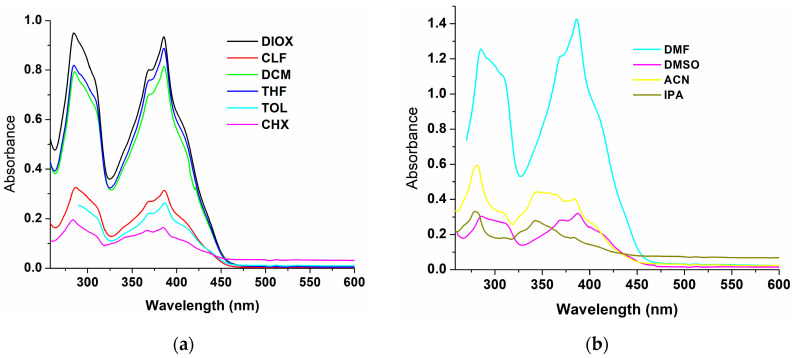
Absorption spectra of the DPyH9 derivative in (**a**) medium- and (**b**) high-polarity solvents.

The UV–vis absorption spectra of DPyH9 have been studied in various solvents with varying polarities to investigate its photophysical behavior. The spectra show two distinct absorption bands, one between 260 and 330 nm and the other at 330–450 nm, with absorption maxima at 284 nm and 386 nm, respectively (Figure 2). These maxima are red-shifted compared to the pyrene monomer (λ_abs_ = 350 nm [27]), indicating an alteration in the electronic structure due to dimerization. The red shift observed in more polar solvents is likely due to the stabilization of the excited state through solvent–solute interactions, enhancing charge transfer.

However, exceptions were found in ACN and IPA, where deviations from the typical absorption maxima were observed (Table 2). Interestingly, DPyH9 exhibits relatively small solvent-dependent variations in its absorption spectra in organic solvents, suggesting that the ground-state electronic structure remains largely stable in these environments [28]. This is likely due to the unique structure of DPyH9, which allows it to maintain its conformation even in the presence of different solvents. The photophysical data for DPyH9 in organic solvents with different polarities are summarized in Table 2.

The Stokes shift values in Table 2 quantify the degree of solvatochromic behavior. The larger shifts in polar solvents (e.g., ACN, IPA) suggest greater stabilization of the excited state via solvent–solute interactions, enhancing charge transfer processes.

**Figure 3:**
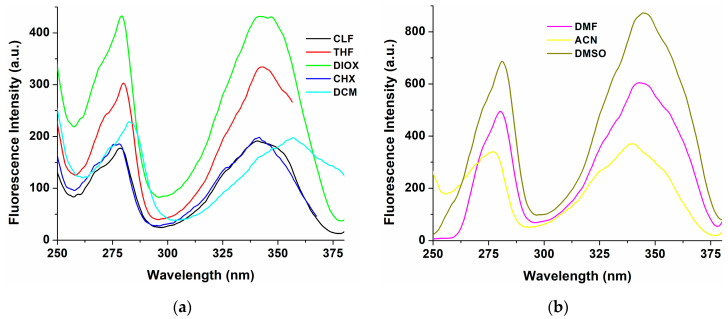

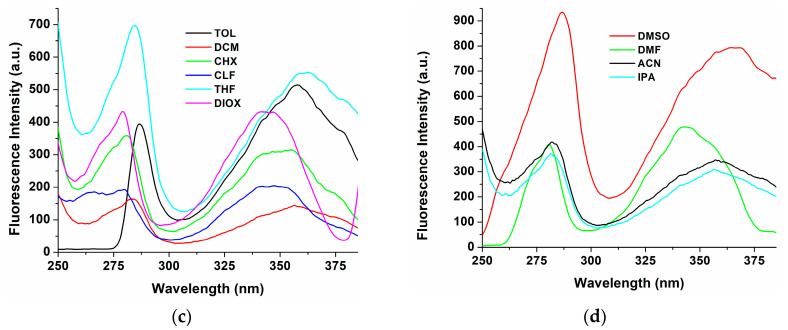
Excitation spectra of DPyH9 monitored at 385 nm (**a**,**b**) and 480 nm or 430 nm (**c**,**d**) wavelengths.

Fluorescence excitation spectra were also studied to evaluate the impact of solvent polarity on energy transfer within DPyH9. The excitation wavelengths of 385 and 430 nm were selected based on emission characteristics specific to the solvents under study. At an excitation wavelength of 385 nm (Figure 3), the excitation and absorption spectra (Figure 2) were similar, indicating minimal solvent–molecule interactions. This similarity suggests that at this excitation wavelength, there were limited effects of the solvent on the energy transfer processes within DPyH9. In contrast, at 430 nm, a broad excitation band was observed, especially in solvents like DCM, indicating that solvent–solute interactions played a more significant role in the energy transfer dynamics at this longer excitation wavelength [29,30].

**Figure 4:**
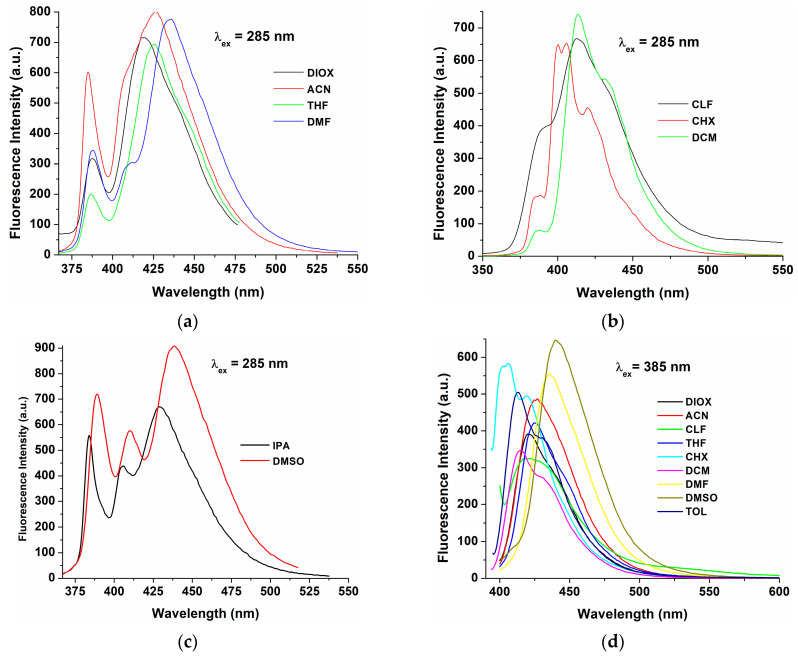
Fluorescence spectra of the DPyH9 derivative in selected solvents of different polarities, excited at 285 nm (**a**–**c**) and 385 nm (**d**).

The fluorescence behavior of DPyH9, a symmetric liquid crystalline pyrene-based imine dimer, was studied in various solvents with different polarities to understand how the solvent environment influences its emission properties. The excitation wavelengths used for studying the fluorescence behavior of DPyH9 were 285 nm (Figure 4a–c) and 385 nm (Figure 4d). The emission spectra of DPyH9 were examined in solvents like DIOX, ACN, DMF, THF, chlorinated solvents, CHX, IPA, and DMSO. The emission profiles varied depending on the solvent used, indicating that the solvent environment significantly impacts the fluorescent characteristics of DPyH9. For instance, in solvents like DIOX, ACN, DMF, and THF (Figure 4a), structured emission profiles with a maximum wavelength (λ_em_,_max_) at 388 nm and a range of 420–435 nm (band II) were observed, indicating specific fluorescence behavior in these polar solvents. In contrast, chlorinated solvents and CHX showed an unclear shoulder in the emission band at 388 nm, suggesting a different fluorescence response compared to the aforementioned polar solvents (Figure 4b). Interestingly, in solvents like IPA and DMSO, three distinct emission peaks were observed (Figure 4c), indicating a more complex fluorescence behavior in these specific environments. The presence of multiple emission peaks suggests the occurrence of different excited state processes in these solvents. The fluorescence spectra of DPyH9 in DMF showed a structured emission band with maxima at 388, 410 ^sh^, and 435 nm. This red shift relative to the pyrene monomer (λ_max_ = 376, 396, and 417 nm [30]) is indicative of enhanced π-electron conjugation in the dimer. Additionally, the observed red shift from nonpolar solvents like CHX (406 nm) to highly polar solvents like DMSO (440 nm) highlights the solvatochromic fluorescence behavior of DPyH9. This behavior can be attributed to the stabilization of the excited state by polar solvents, leading to greater charge transfer within the molecule.

**Figure 5:**
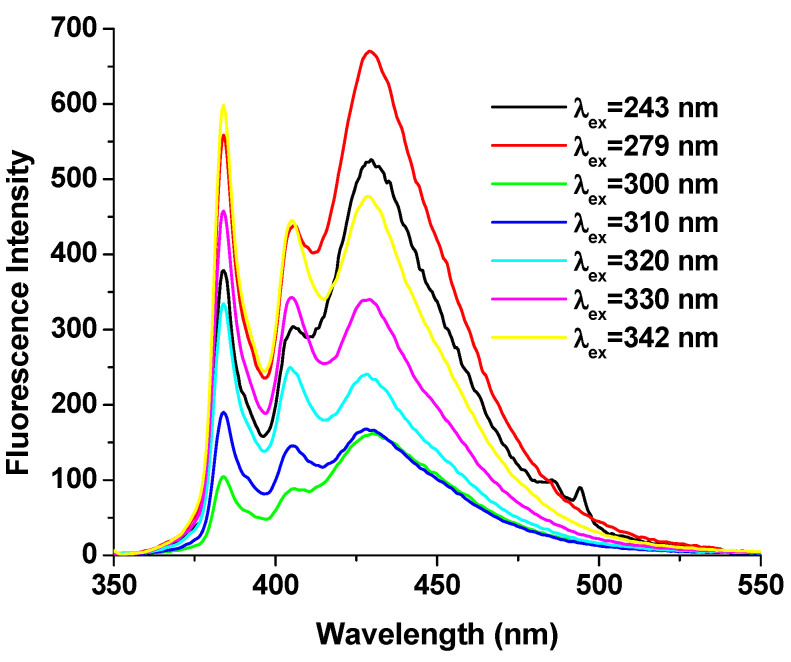
Excitation-independent emission spectra of DPyH9 in the IPA solvent.

The emission spectra showed excitation wavelength-independent fluorescence emission behavior at excitation wavelengths ranging from 285 to 385 nm (Figure 5). The wavelength-independent emission was coupled with intensity variations unrelated to the excitation wavelength. This behavior was attributed to the presence of defects in the pyrene imine dimer structure crystals, which follow the fundamental rules of excited state emission [31].

### 2.2. Acidochromic Behavior

**Figure 6:**
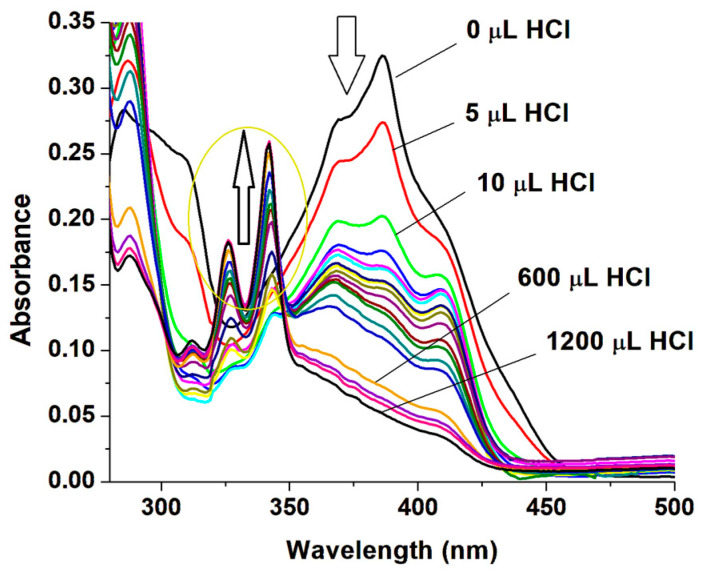
UV-vis absorption spectra upon protonation of DPyH9 pyrene imine dimer by HCl in DMF. Arrows indicate changes in absorbance upon HCl addition.

Having established that DPyH9 exhibits distinct photophysical responses, including solvent-dependent absorption and fluorescence changes—we next evaluated its responsiveness to chemical stimuli. First, the acidochromic behavior was examined by monitoring spectral changes upon HCl addition. The acidochromic properties of DPyH9 were investigated by monitoring changes in its absorption spectra upon the addition of HCl (Figure 6). The introduction of HCl resulted in notable spectral changes, including the emergence of intense absorption bands in the 300–350 nm range and a significant reduction or complete disappearance of the broad band initially observed between 350 and 450 nm. Furthermore, the broad band centered at 380 nm disappeared completely upon the addition of excess HCl. These spectral transformations indicate a modification in the electronic structure of DPyH9 upon interaction with HCl. The observed changes can be attributed to the protonation of the imine nitrogen [31,32] within the pyrene-based imine dimer. This response highlights the potential of DPyH9 as a candidate for acidochromic sensing applications.

### 2.3. Metal Ion Sensing Behavior

**Figure 7:**
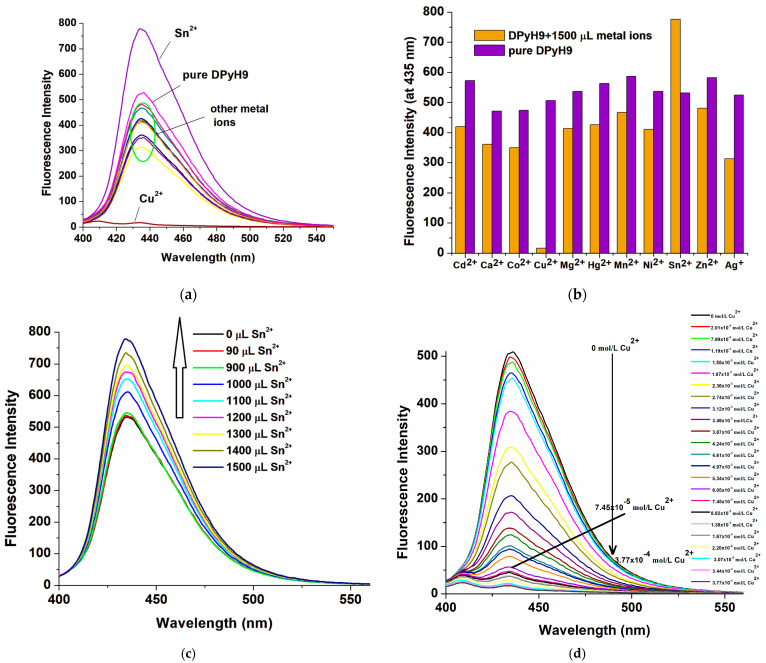
(**a**) Fluorescence emission spectra of DPyH9 upon addition of 1500 μL of various metal ions (Cd^2+^, Ca^2+^, Co^2+^, Cu^2+^, Mg^2+^, Hg^2+^, Mn^2+^, Ni^2+^, Sn^2+^, Zn^2+^, and Ag^+^); (**b**) bar chart showing the relative fluorescence intensity (at 435 nm) changes in DPyH9 upon addition of 1500 μL of various metal ions; (**c**) fluorescence spectrometric titration of DPyH9 with Sn^2+^; (**d**) fluorescence spectrometric titration of DPyH9 with Cu^2+^ (λ_exc_ = 385 nm, DMF). Arrows indicate changes in signal intensity with increasing metal ion concentrations.

The fluorescence properties of pyrene-based compounds for metal ion detection have been extensively explored, showing distinct responses to various metal ions [8,33,34]. The metal ion sensing ability of the pyrene imine dimer DPyH9 was investigated in DMF by screening a range of metal ions, including Cd^2+^, Ca^2+^, Co^2+^, Cu^2+^, Mg^2+^, Hg^2+^, Mn^2+^, Ni^2+^, Sn^2+^, Zn^2+^, and Ag^+^, by adding 1500 μL of each ion to the solution. Among the metal ions tested, Sn^2+^ and Cu^2+^ showed the most distinct responses, prompting further investigation into their interactions with DPyH9. The observed quenching efficiencies varied for different metal ions. Notably, the quenching effects were less pronounced for Ca^2+^, Cd^2+^, Co^2+^, Mg^2+^, Hg^2+^, Mn^2+^, Ni^2+^, Zn^2+^, and Ag^+^, except for Sn^2+^ and Cu^2+^ (Figure 7a,b). The gradual addition of Sn^2+^ (0–900 μL) induced a weak fluorescence response compared with the initial fluorescence of pure DPyH9. However, at 900 μL of Sn^2+^ (Figure 7c), a noticeable enhancement in fluorescence intensity was observed, indicating a distinct interaction between DPyH9 and Sn^2+^ ions. This enhancement can be attributed to the inhibition of C=N isomerization and the formation of a stable chelate complex with Sn^2+^, leading to an extended π-electron conjugated system.

On the other hand, the presence of Cu^2+^ ions caused a significant reduction in fluorescence, with a maximum quenching efficiency of up to 96% with saturation around 600 μL (1.94 × 10^−4^ mol/L, Figure 7d).

To quantitatively assess the binding interactions observed during the fluorescence titrations, we applied the Benesi–Hildebrand method. This approach relates changes in fluorescence intensity to the concentration of the added metal ion and enables the calculation of the binding constant (K_a_) using the following equation [35]:

1/(F − F_0_) = 1/[K_a_(F_max_ − F_0_)[M^n+^]] + 1/(F_max_ − F_0_) (1)

1/(F_0_ − F) = 1/[K_a_(F_0_ − F_max_)[M^n+^]^2^] + 1/(F_0_ − F_max_) (2)

where F_0_, F_max_, and F represent fluorescence intensities without any M^n+^, with added [M^n+^]_max_ and after the addition of a given amount of M^n+^ to a given concentration.

**Figure 8:**
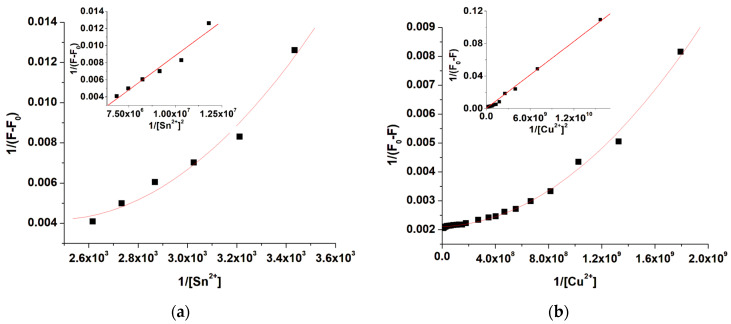
Benesi–Hildebrand plots for the determination of the binding constant of DPyH9 with (**a**) Sn^2+^ and (**b**) Cu^2+^ ions. Insets show plots of 1/(F − F_0_) or 1/(F_0_ − F) vs. 1/[M^n+^]^2^.

The K_a_ values were calculated from the slope and intercept of the straight line of the inset plots of 1/(F − F_0_) vs. 1/[M^n+^]^2^ (Figure 8a,b) and were determined to be 4.51 × 10^6^ M^−1^ for Sn^2+^ and 4.03 × 10^7^ M^−1^ for Cu^2+^, respectively. The higher K_a_ value for Cu^2+^ indicates a stronger interaction between DPyH9 and these metal ions and suggests a more stable complex formation, making it a more sensitive sensor for this metal ion. DPyH9 exhibited a 1:2 stoichiometry with both Sn^2^⁺ and Cu^2^⁺, as indicated by nonlinear Benesi–Hildebrand plots (Figure 8). The higher binding constant for Cu^2^⁺ (4.03 × 10⁷ M⁻^1^) suggests stronger complexation, while the lower detection limit for Sn^2^⁺ (1.61 × 10⁻⁵ M) reflects its enhanced sensitivity despite weaker binding.

The fluorescence response of DPyH9 to metal ions can be attributed to complex formation, as also reported in previous studies that explore combined sensing and mechanistic approaches [36,37]. Also, the binding constants offer further insights into the interactions. The higher binding constant (K_a_) for Cu^2^⁺ (4.03 × 10⁷ M⁻^1^) compared to Sn^2^⁺ (4.51 × 10⁶ M⁻^1^) suggests a stronger coordination with Cu^2^⁺, likely involving multiple binding sites. Based on this, we propose that Sn^2^⁺ binds to the imine nitrogen and ether oxygen atoms, forming a tetrahedral complex. This coordination weakens the C=N isomerization, stabilizing the excited state and leading to enhanced fluorescence at higher concentrations, similar to what has been reported for other pyrene-Schiff base systems [9]. On the other hand, Cu^2^⁺ is expected to coordinate with two imine nitrogen atoms, forming either a square planar or octahedral complex [38]. This interaction would rigidify the structure, allowing for efficient photoinduced electron transfer (PET) and subsequent quenching, in line with Cu^2^⁺’s well-known affinity for imine ligands.

The limit of detection (LOD) represents the lowest concentration of metal ions that can be reliably detected under experimental conditions. In this study, LOD was determined using the equation LOD = K × SD/S [39], where K = 3, SD is the standard deviation of the blank solution, and S is the slope of the fluorescence intensity vs. metal ion concentration curves. The LOD values were calculated to be 1.61 × 10^−5^ M for Sn^2+^ and 4.73 × 10^−5^ M for Cu^2+^, respectively.

The detection limit and binding constant obtained in the present study were compared with the reported pyrene derivative Sn^2^⁺ and Cu^2^⁺ fluorescent sensors (Table 3). Although Cu^2^⁺ exhibits a higher binding constant (K_a_ = 4.03 × 10⁷ M⁻^1^) compared to Sn^2^⁺ (K_a_ = 4.51 × 10⁶ M⁻^1^), the detection limit for Sn^2^⁺ (LOD = 1.61 × 10⁻⁵ M) is lower than that for Cu^2^⁺ (LOD = 4.73 × 10⁻⁵ M). This is attributed to the distinct fluorescence response mechanisms: while Cu^2^⁺ induces strong static fluorescence quenching, Sn^2^⁺ exhibits an initial fluorescence decrease followed by an enhancement at higher concentrations. The partial fluorescence recovery in the presence of Sn^2^⁺ results in a higher signal-to-noise ratio at low concentrations, enabling improved sensitivity and a lower detection limit. The significantly higher binding constant (K_a_) for Cu^2^⁺ suggests stronger complexation, while the lower detection limit indicates enhanced sensitivity compared to existing sensors. These results suggest that DPyH9 shows promising sensitivity and a strong binding affinity towards the Sn^2^⁺ and Cu^2^⁺ metal ions.

## 3. Materials and Methods

### 3.1. Materials

1-Aminopyrene, N,N-dimethylformamide (DMF), and ethanol were purchased from Sigma-Aldrich (Merck KGaA, Darmstadt, Germany) and used as received. The precursor 1,9-bis(4-formylphenoxy)-nonane was prepared according to published procedures [43,44]. The solvents used in the experiments, such as CHX (cyclohexane), TOL (toluene), DIOX (1,4-dioxane), THF (tetrahydrofuran), CLF (chloroform), DCM (dichloromethane), DMF, DMSO (dimethyl sulfoxide), ACN (acetonitrile), and IPA (2-propanol), were of spectroscopic grade and were used without any additional purification.

### 3.2. Synthesis and Characterization of 1,9-bis[4-(Pyreneiminomethylidene)-phenoxy]-nonane [15]

**Scheme 1:**
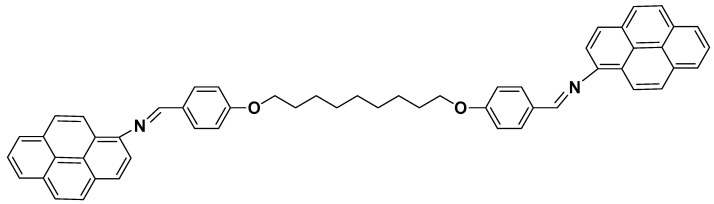
Structure of the investigated compound (DPyH9).

In a three-necked flask equipped with a magnetic stirrer and a nitrogen system, 0.15 g of 1,9-bis(4-formylphenoxy)-nonane, 0.2 g of 1-aminopyrene, and 2.2 mL of DMF were added. The reaction mixture was refluxed for 60 h under a nitrogen atmosphere. After 12 h, a few drops of acetic acid were added as a catalyst. Finally, the precipitated product was filtered, washed several times with ethanol, and dried in a vacuum for 24 h at 60 °C to obtain the desired dimer (Scheme 1).

Characterization data:

FTIR (KBr, ν, cm^–1^): 3038 (=C–H stretch of the aromatic rings), 2933–2851 (C–H stretch of aliphatic chains), 1627 (–N=CH– stretch), 1601, 1587, 1511 (C–C ring stretch), 1243, 1163 (C–O–C– stretch), 843 (absorption band assigned to 1,4-phenylene ring). ^1^H-NMR (d6-DMSO, δ, ppm): 8.82 (s, 2H, N=CH), 7.16, 7.14 (d, 4H), 8.10, 8.08 (d, 4H), 7.92, 7.90 (d, 2H), 8.05, 8.03 (d, 2H), 8.12, 8.10 (d, 2H), 8.17, 8.15 (d, 2H), 8.19, 8.17 (d, 2H), 8.27, 8.25 (d, 2H), 8.29, 8.27 (d, 2H), 8.31, 8.29 (d, 2H), 8.65, 8.63 (d, 2H), 4.12–4.09 (t, 4H, α to Ar–O–), 1.80–1.75 (m, 4H, β to Ar–O–), 1.48–1.38 (m, 10H, γ, δ, and ε to Ar–O–).

### 3.3. Characterization Methods

Infrared spectra were recorded on an FTIR Bruker Vertex 70 spectrophotometer (Ettlingen, Germany) in transmission mode at wavenumbers ranging from 400 cm^−1^ to 4000 cm^−1^. The samples were mixed with KBr and pressed into pellet form.

Proton nuclear magnetic resonance spectra were recorded with a Bruker Advance DRX 400 MHz spectrometer (Rheinstetten, Germany) equipped with a 5 mm, direct detection, multinuclear probe. Chemical shifts were reported in δ units (ppm) relative to the residual peak of the solvent.

Polarized light optical microscopy (PLM) observations were carried out with a Zeiss Axio Imager M2 microscope, Carl Zeiss AG, (Wetzlar, Germany). The sample was prepared by casting a small amount on a glass plate, which was subjected to two heating–cooling cycles using a scan rate of 10 °C/min. For all measurements, a 50× objective lens was used. The eyepiece had 10× magnification.

The differential scanning calorimetry (DSC) analysis was performed using a Pyris Diamond DSC, Perkin Elmer system (Shelton, CT, USA), under a nitrogen atmosphere. It recorded two heating–cooling cycles using a scan rate of 10 °C/min. The phase transition temperatures were taken as the maximum of the endothermic and exothermic peaks.

UV-vis absorption spectra were performed using an Analytic Jena 210+ spectrophotometer (Jena, Germany), and steady-state emission and excitation spectra were recorded using a Perkin Elmer LS55 fluorescence spectrophotometer. Quartz cuvettes with a pathlength of 1 cm were used for fluorescence and UV-vis absorption data acquisition. These measurements were performed at room temperature. The acidochromic behavior of the DPyH9 derivative was evaluated by titrating DPyH9 in a DMSO solution with incremental additions of 0.1 N HCl (hydrochloric acid) and measuring absorption spectra after stabilization. Titrations of DPyH9 (2.5 mL) against metal ion solutions were performed using a Hamilton microsyringe (Hamilton, Reno, Nev.), and the absorption and fluorescence spectra were measured after each addition of metal ions. Stock solutions (1.07 × 10^−3^ mol/L) of chloride or sulfate salts of different metals (CuCl_2_ × 6H_2_O, AgNO_3_, CoCl_2_ × 6H_2_O, NiCl_2_ × 6H_2_O, HgCl_2_, CaCl_2_, MgCl_2_ × 6H_2_O, SnCl_2_ × 2H_2_O, ZnSO_4_ × 7H_2_O, CdSO_4_, and MnSO_4_ × H_2_O) were prepared in DMF solution.

## 4. Conclusions

This study investigated the solvatochromic, acidochromic, and metal ion sensing behavior of a pyrene-based imine dimer (DPyH9). In summary, DPyH9 demonstrates unique liquid crystalline behavior, solvatochromic fluorescence, and selective metal ion sensing capabilities, particularly towards Sn^2+^ and Cu^2+^, making it a promising candidate for sensing applications. Additionally, DPyH9 demonstrates excitation wavelength-independent fluorescence emission behavior, with the fluorescence spectra varying significantly depending on the solvent used. This variation indicates a notable impact of the solvent environment on the fluorescence characteristics. Moreover, DPyH9 showed solvatochromic fluorescence behavior, with a red shift in the emission spectra observed in more polar solvents. The compound also exhibited acidochromic behavior, as evidenced by changes in its absorption spectra upon the addition of hydrochloric acid. DPyH9 can be used for metal ion sensing, displaying distinct responses to Sn^2+^ and Cu^2+^ ions. The binding constant (K_a_) for Cu^2+^ (4.03 × 10^7^ M^−1^) was significantly higher than that for Sn^2+^ (4.51 × 10^6^ M^−1^), indicating a stronger interaction between DPyH9 and Cu^2+^. Additionally, the detection limit for Cu^2+^ (4.73 × 10^−5^ M) was lower than that for Sn^2+^ (1.61 × 10^−5^ M), suggesting enhanced sensitivity for Cu^2+^ detection. DPyH9’s acidochromic response, solvatochromic fluorescence, and selective detection of Sn^2^⁺ and Cu^2^⁺ underscore its potential as a multifunctional sensor. The compound’s higher sensitivity to Sn^2^⁺ (LOD = 1.61 × 10⁻⁵ M) compared to Cu^2^⁺ (LOD = 4.73 × 10⁻⁵ M) aligns with its dual fluorescence response, enabling nuanced ion discrimination.

## Figures and Tables

**Table 1:** Transition temperatures (°C) and enthalpy changes (KJ mg^–1^) of DPyH9.

	PLM ^a^		DSC ^b^	
	1st Scan	2nd Scan	1st Scan	2nd Scan
Heating	Cr 136 I	– 50 N 102 I	Cr 134 (49.77) ^d^ I	G 43 N 100 (0.50) I
Cooling	I 108 N – ^c^	I 98 N –	I 98 (0.54) N 38 G	I 98 (0.55) N 36 G

Abbreviations: Cr—crystalline; N—nematic; I—isotropic; G—glass transition; ^a^ transition temperature resulted from polarized light optical microscopy information; ^b^ transition temperatures recorded by differential scanning calorimetry; ^c^ frozen phase; ^d^ enthalpy value (in brackets).

**Table 2:** Spectroscopic parameters of DPyH9 in organic solvents with different polarities.

Solvents	λ_abs,max_ (nm)	Δλ ^(a)^ (nm)	λ_em,max_ (nm)	
			λ_ex_ = 285 nm	λ_ex_ = 385 nm
CHX	284, 385	21	400, 406, 420 ^sh^	401 ^sh^, 406, 419 ^sh^
TOL	311 ^sh^, 386	27	-	413, 434 ^sh^
DIOX	285, 309 ^sh^, 369 ^sh^, 386	33	388, 419	420
THF	285, 309 ^sh^, 369 ^sh^, 386	41	387, 427	425
CLF	286, 309 ^sh^, 369 ^sh^, 386	27	390 ^sh^, 413	421
DCM	285, 309 ^sh^, 369 ^sh^, 386	27	413, 431 ^sh^	415, 431 ^sh^
DMF	285, 309 ^sh^, 368 ^sh^, 386	49	388, 410 ^sh^, 435	435
DMSO	284, 312 ^sh^, 369 ^sh^, 388	51	390 ^sh^, 410, 439	440
ACN	281, 347, 384 ^sh^	80	388, 427	427
IPA	280, 343	86	384, 405, 429	384, 405, 429 ^(b)^

Abbreviations: λ_abs,max—_maximum absorption wavelength; λ_ex—_maximum excitation wavelength; λ_em,max—_maximum emission wavelength; CHX—cyclohexane; TOL—toluene; DIOX—1,4-dioxane; THF—tetrahydrofuran; CLF—chloroform; DCM—dichloromethane; DMF—N,N-dimethylformamide; DMSO—dimethyl sulfoxide; ACN—acetonitrile; IPA—2-propanol; ^(a)^ Δλ = λ_em,max_ − λ_abs,max_ − Stoke shifts: ^(b)^ results for λex − 343 nm; ^sh^—shoulder.

**Table 3:** Performance of the DPyH9 sensor in terms of its detection limit and binding constant in comparison to previously reported pyrene derivative sensors for Sn^2^⁺ and Cu^2^⁺.

Sensors/Metal Ions	LOD	Association (Binding) Constant	Refs.
Pyrene-appended Schiff base/Cu^2^⁺	219 nM	4.95 × 10^−6^ M^−1^	[40]
1(pyrene-1-ylmethyl)-L-phenylalanine/Cu^2^⁺	2 × 10^−8^ M	5 × 10^4^ M^−1^	[41]
Fluorescent probe derived from Pyrene/Cu^2+^	0.16 μM	6.5 × 10^5^ M^−1^	[42]
Schiff base PY-SB/Sn^2+^	5.4 µM	2 × 10^4^ M^−1^	[9]
Pyrene-oligo(azomethine)/Sn^2+^	4.24 nM	2.40 × 10^4^ M^−1^	[8]
DPyH9/Cu^2+^	4.73 × 10^−5^ M	4.03 × 10^7^ M^−1^	this work
DPyH9/Sn^2+^	1.61 × 10^−5^ M	4.51 × 10^6^ M^−1^	this work

## Data Availability

Data are contained within the article.
